# The histone methyltransferase DOT1L is a new epigenetic regulator of pulmonary fibrosis

**DOI:** 10.1038/s41419-021-04365-5

**Published:** 2022-01-17

**Authors:** Di Yang, Peng Xu, Haibi Su, Wen Zhong, Jie Xu, Zhenghua Su, Xinhua Liu

**Affiliations:** 1 Pharmacophenomics Laboratory, Human Phenome Institute, Fudan University, 201203 Shanghai, China; 2 Department of Pharmacy, Taihe Hospital, Hubei University of Medicine, Shiyan, Hubei China

**Keywords:** Epigenetics, Respiratory tract diseases

## Abstract

Idiopathic pulmonary fibrosis (IPF) is a progressive interstitial lung disease with increasing occurrence, high death rates, and unfavorable treatment regimens. The pathogenesis underlying IPF is complex and the epigenetic contributions to IPF are largely unknown. Recent studies have shown that DOT1L (Disruptor of telomeric silencing-1 like), a histone H3K79 methyltransferase, contributes to fibrosis response, but its role in IPF remains unclear. DOT1L, H3K79me3, and the profibrotic proteins levels were upregulated in the pulmonary fibrosis models both in vivo and in vitro. Lentivirus-mediated DOT1L knockdown or DOT1L-specific inhibitor EPZ5676 alleviated the pathogenesis of bleomycin-induced mouse pulmonary fibrosis. Furthermore, heterozygous DOT1L-deficient mice (*Dot1l*^+/−^) showed less sensitive to pulmonary fibrosis, as shown by decreased lung fibrosis phenotypes in vivo. Mechanically, DOT1L regulated TGF-β1-induced fibroblasts fibrosis by increasing enrichments of H3K79me3 on the promoter of *Jag1* gene (encoding the Notch ligand Jagged1), enhancing the expression of Jagged1, which in turn stimulated exuberant Notch signaling and actuated the fibrosis response. In conclusion, our study confirmed DOT1L to be an epigenetic modifier in the pathogenesis of lung fibrosis, revealed a counterbalancing mechanism governing *Jag1* transcription by modulating H3K79 trimethylation at the *Jag1* promoter, activating the Notch signaling, and affecting the expression of profibrotic proteins to accelerate the lung fibrosis.

## Introduction

Idiopathic pulmonary fibrosis (IPF) is a chronic, progressive, and usually fatal fibrotic disease of the lung, characterized by extreme deposition of extracellular matrix (ECM) and destruction of the normal lung architecture [1]. The median survival from diagnosis is estimated as 3–5 years [2]. The most important known prognostic determinants for mortality are decline in lung function, acute exacerbations as well as pulmonary hypertension [3, 4]. Still, despite extensive research over the past 25 years, treatment options for IPF are limited to two recently approved drugs that slow down disease progression [5, 6]. Hence, we are therefore in need of prevention and additional treatment strategies for this fatal lung disease.

Pulmonary fibrosis is derived by initially repeated epithelial cell damage and subsequently progressive lung scarring that results in the formation of fibroblast and myofibroblast foci [7]. These foci secrete excessive ECM that is mainly composed of collagens, depositing in the lung interstitium and destructing the lung structure, which ultimately leads to respiratory failure and death [8]. Due to the complicated pathogenesis of pulmonary fibrosis, better identification of reliable biomarkers in the pulmonary fibrosis is needed to recognize new therapeutic targets for IPF diseases.

Epigenetic regulation has been well recognized as a key mechanism in activating or silencing gene expression levels and has implications in the occurrence and development of IPF, especially given the association of IPF with cigarette smoking and the relationship between cigarette smoke and changes in DNA methylation, histone modifications and miRNAs [9]. The Disruptor of telomeric silencing 1-like (DOT1L) is currently the only identified histone methyltransferase that methylates lysine-79 of histone H3 (H3K79) in mammals and is involved in epigenetic regulation of gene transcription [10, 11]. DOT1L has been reported to play a significant role in DNA damage/repair, cardiac function and carcinogenesis [12]. Quantitative chromatin immunoprecipitation studies of H3K79 methylation across the human genome reveal that H3K79me and H3K79me2 are linked to active gene transcription [13, 14]. This histone methylation typically functions in transcriptional regulation [15, 16], telomeric silencing [17], cell-cycle regulation [18], and DNA damage repair [19]. It has been reported that DOT1L is downregulated in dilated cardiomyopathy and its cardiac-specific deletion results in the global loss of H3K79me2/3 [20]. However, whether DOT1L is responsible for pulmonary fibrosis has yet to be fully elucidated, and DOT1L-based approaches may be critical for discovering novel anti-fibrotic therapies, therefore improving clinical outcomes for IPF patients.

The Notch signaling represents an evolutionarily conserved signaling system with essential roles in mediating key cellular processes in developmental and differentiated tissues [21, 22]. It is now clear that the Notch family of transmembrane receptors (Notch1–4) and Notch ligands including Jagged1, Jagged2, Dll1, and Dll4 were found in mammals [23]. Upon ligand binding, the Notch intracellular domain (NICD) is released by proteolytic cleavage, then translocate into the nucleus and regulates Notch-dependent gene cohorts [24, 25]. It has been well documented that Jagged1/Notch-signaling increases and is essential for myofibroblast differentiation in the bleomycin model [26]. Besides, repeated lung injury also stimulates Wnt/β-catenin-dependent persistent upregulation of the Notch ligand Jagged1 (encoded by *Jag1*) expression which in turn stimulates exuberant Notch signaling in perivascular fibroblasts and enhances fibrosis [27].

Bleomycin-induced lung injury and subsequent fibrosis in mice is a well-characterized histological and biochemical model of pulmonary fibrosis to decipher the role of specific factors in the development of the disease [28, 29]. Transforming growth factor (TGF)-β1 is a key profibrotic factor to induce the differentiation process of fibroblasts into myofibroblasts, releasing abundant cytokines and ECM to enlarge the fibrosis foci. Therefore, the current study is aimed to investigate the possible effects of DOT1L on lung fibrosis and inflammation responses both in TGF-β1-induced fibroblasts and in bleomycin-induced mouse models of IPF. Our study identifies DOT1L as a epigenetic regulator of lung fibrosis. Blockade of DOT1L preserves pulmonary fibrosis by preventing the hyper-activation of Jagged1-Notch signaling. Overall, our data demonstrate the importance of DOT1L in maintaining lung health and provide a rationale for potential therapeutic interventions in the treatment of pulmonary fibrosis.

## Results

### The expression of DOT1L increases in pulmonary fibrosis models both in vivo and in vitro

**Fig. 1:**
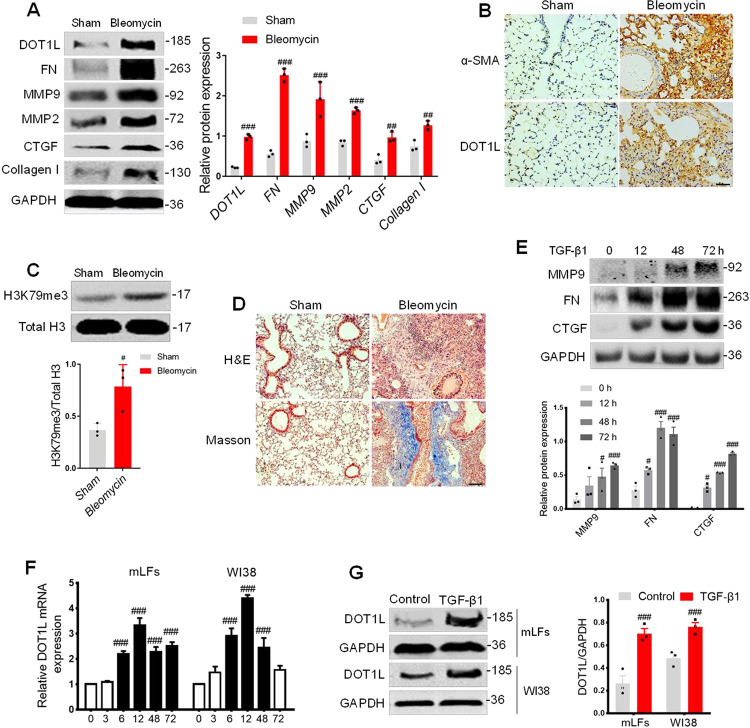
DOT1L is increased in pulmonary fibrosis models both in vivo and in vitro. **A**–**D** DOT1L is increased in pulmonary fibrosis models in vivo. Western blot analysis of DOT1L and fibrosis markers (FN, MMP9, MMP2, CTGF, and Collagen I) in lung tissues of bleomycin-administrated mice compared to sham mice (**A**); representative immunohistochemistry staining of α-SMA and DOT1L in the lung sections from the sham and bleomycin-induced pulmonary fibrosis groups. Scale bars: 50 μm (**B**); H3K79me3 levels in lung tissues (**C**); representative hematoxylin and eosin (H&E) and Masson’s trichrome staining of lung sections from the sham and bleomycin-induced pulmonary fibrosis groups. Scale bars: 100 μm (**D**); all data are presented as mean ± S.D., *n* = 6–10/group, ^#^*p* < 0.05, ^##^*p* < 0.01, ^###^*p* < 0.001 compared with sham. **E**–**G** DOT1L is increased in pulmonary fibrosis models in vitro. Immunoblot analyzed for MMP9, FN, and CTGF in TGF-β1-induced mLFs (**E**); mRNA and protein levels of DOT1L increased in both TGF-β1-induced mLFs and WI38 cells (**F**, **G**); all data are presented as mean ± S.D. of three independent experiments. ^#^*p* < 0.05, ^##^*p* < 0.01, ^###^*p* < 0.001 compared with control.

To investigate the role of DOT1L in pulmonary fibrosis, we constructed the widely used mouse pulmonary fibrosis model by intratracheal administration of bleomycin. The results showed that levels of fibrosis markers (fibronectin (FN), matrix metalloproteinase 9 (MMP9), MMP2, connective tissue growth factor (CTGF), and Collagen I) significantly increased in bleomycin-induced lung fibrosis. Interestingly, DOT1L was also upregulated during pulmonary fibrosis (Fig. 1A). Similarly, both DOT1L and alpha-smooth muscle actin (α-SMA) showed significantly increased expression levels in bleomycin-induced lung fibrosis, as detected by immunohistochemical staining (Fig. 1B). Consistent with the elevated expression of DOT1L, the abundance of trimethylation of H3K79 (H3K79me3) in the lung fibrosis also increased relative to total histone H3 levels (Fig. 1C). Furthermore, the morphometric analysis of the fibrotic lung sections with hematoxylin and eosin (H&E) or Masson’s trichrome staining showed ECM deposition, alveolar structural disorder, and fibrous thickening around the alveoli and trachea compared to the normal lung tissue (Fig. 1D). These above data suggest that DOT1L is associated with pulmonary fibrosis in vivo.

To further address the correlation between DOT1L and pulmonary fibrosis in vitro, we used TGF-β1-induced pulmonary fibroblast transformation. The results showed that fibrosis-related proteins such as MMP9, FN, and CTGF increased in a time-dependent manner in TGF-β1-treated primary mouse lung fibroblasts (mLFs; Fig. 1E). Interestingly, the mRNA and protein levels of DOT1L were upregulated in both TGF-β1-induced mLFs and WI38 cells (Fig. 1F, G). These results support the idea that DOT1L is associated with pulmonary fibrosis not only in bleomycin-induced mice model but also in TGF-β1-induced cell model.

### DOT1L disruption inhibits bleomycin-induced mouse pulmonary fibrosis in vivo

**Fig. 2:**
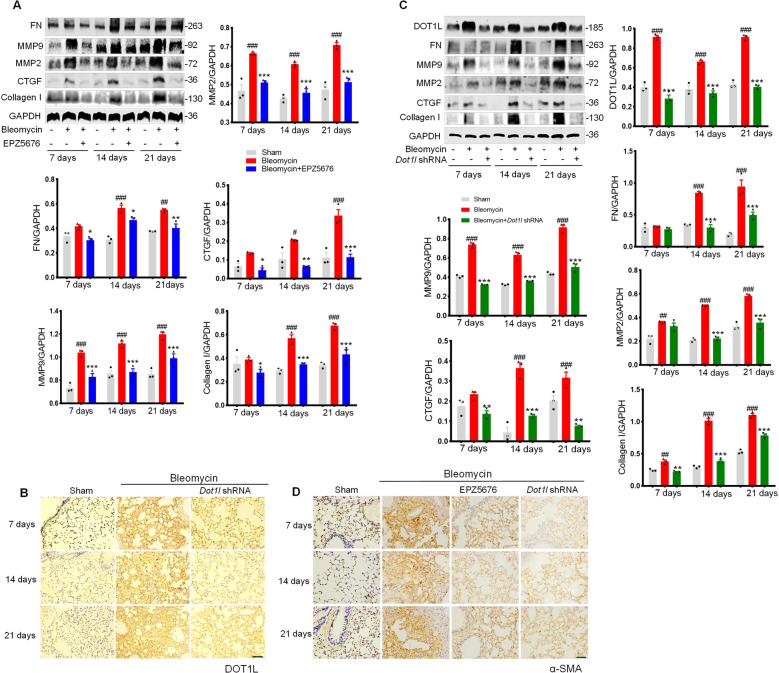
DOT1L disruption inhibits bleomycin-induced mouse pulmonary fibrosis in vivo. **A** The expression levels of FN, MMP9, MMP2, CTGF, and Collagen I were examined by western blot in bleomycin-induced mice after treatment with EPZ5676 for 7, 14, and 21 days, respectively. **B** Representative immunohistochemistry staining of DOT1L in the lung sections from bleomycin-induced mice after treatment with *Dot1l* shRNA for 7, 14, and 21 days, respectively. Scale bars: 50 μm. **C** Immunoblot analysis of DOT1L and fibrosis-associated proteins (FN, MMP9/2, CTGF, and Collagen I) in bleomycin-induced mice after treatment with *Dot1l* shRNA for 7, 14, and 21 days, respectively. **D** Representative immunohistochemistry staining of α-SMA in the lung sections from bleomycin-induced mice after treatment with EPZ5676 or *Dot1l* shRNA for 7, 14, and 21 days, respectively. Scale bars: 50 μm. All data are presented as mean ± S.D.; *n* = 6–10/group, ^#^*p* < 0.05, ^##^*p* < 0.01, ^###^*p* < 0.001 compared with the sham group; *p < 0.05, **p < 0.01, ***p < 0.001 compared with the bleomycin group.

To further study the regulation of DOT1L in pulmonary fibrosis, the small-molecule inhibitor of DOT1L (EPZ5676) was used to inhibit the DOT1L activity. First, we examined fibrotic responses after treatment with EPZ5676. Notably, EPZ5676 suppressed the expression levels of FN, MMP9/2, CTGF, and Collagen I after bleomycin administration for 7, 14, and 21 days, respectively (Fig. 2A). In parallel, we also silenced DOT1L expression by using lentivirus-mediated knockdown of DOT1L (*Dot1l* short hairpin RNA (shRNA)) in mice model. As shown in Fig. 2B, *Dot1l* shRNA decreased DOT1L expression demonstrated by immunohistochemistry. As expected, the protein expression levels of FN, MMP9/2, CTGF, and Collagen I also decreased by *Dot1l* shRNA in lung fibrosis process (Fig. 2C). Furthermore, both EPZ5676 and *Dot1l* shRNA reduced α-SMA expression, as indicated by immunohistochemistry staining (Fig. 2D).

**Fig. 3:**
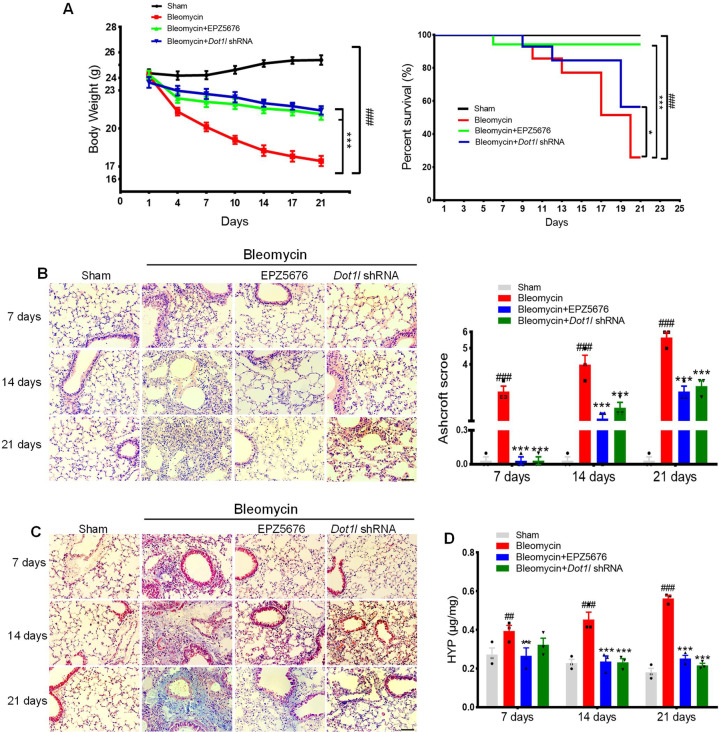
DOT1L is critical for the pathology of pulmonary fibrosis. **A** The body weight (left) and survival rate (right) of mice decreased in bleomycin-administrated mice after *Dot1l* shRNA lentivirus or EPZ5676 treatment for 7, 14, and 21 days. **B** H&E staining and Ashcroft histopathological grading score are measured on days 7, 14, and 21 in bleomycin-administrated mice after *Dot1l* shRNA lentivirus and EPZ5676 treatment, scale bars: 50 μm. **C** Representative images of Masson’s trichrome staining of lung sections, scale bars: 50 μm. **D** Levels of collagen deposition in the lung are determined by hydroxyproline estimation. All data are presented as mean ± S.D.; *n* = 6–10/group, ^##^*p* < 0.01, ^###^*p* < 0.001 compared with the sham group; *p < 0.05, **p < 0.01, ***p < 0.001 compared with the bleomycin group.

In addition, mice treated with EPZ5676 or *Dot1l* shRNA lentivirus preserved the mice body weight and survival rate upon bleomycin administration (Fig. 3A) and further maintained relatively complete lung tissues without fibrosis foci and lower Ashcroft histopathological grading score compared with the bleomycin group (Fig. 3B). Meanwhile, the Masson’s trichrome staining and the hydroxyproline content results indicated that the collagen deposition increased after bleomycin administration, which were reversed by either disruption of DOT1L with shRNA or inhibition of DOT1L with small-molecule inhibitor EPZ5676 (Fig. 3C, D). Taken together, these above results establish for the first time that DOT1L is critical for the pathology of pulmonary fibrosis, and disruption of DOT1L suppresses bleomycin-induced lung fibrosis and injury in vivo.

### Heterozygous DOT1L-deficient mice show less sensitive to the development of pulmonary fibrosis

**Fig. 4:**
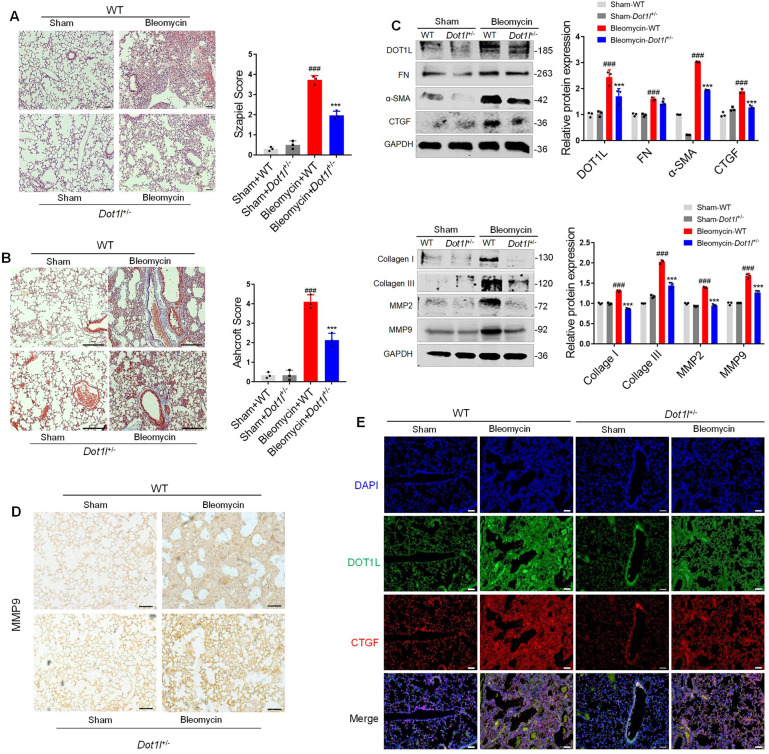
DOT1L heterozygote mice is insensitive to the development of pulmonary fibrosis. WT and *Dot1l*^+/−^ mice were subjected to bleomycin for 14 days. **A** Representative hematoxylin and eosin (H&E) staining of lung sections from bleomycin-induced wild-type (WT) or *Dot1l*^+/−^ mice, scale bars: 20 μm. The detailed Szapiel score is shown on the right panel. **B** Representative Masson’s trichrome staining of lung sections from bleomycin-induced wild-type (WT) or *Dot1l*^+/−^ mice, scale bars: 100 μm. The pulmonary fibrosis stained by Masson’s trichrome staining was evaluated according to the Ashcroft score on the right panel. **C** Expression levels of pulmonary fibrosis markers (FN, CTGF, α-SMA, Collagen I/III, and MMP2/9) in lung tissue of bleomycin-induced WT or *Dot1l*^+/−^ mice, as detected by western blot. **D** Representative immunohistochemistry staining of MMP9 in the lung sections from bleomycin-induced WT or *Dot1l*^+/−^ mice, scale bars: 50 μm. **E** The immunofluorescence double staining with anti-DOT1L and anti-CTGF in lung sections of bleomycin-induced WT or *Dot1l*^+/−^ mice, scale bars: 20 μm. All data are presented as mean ± S.D.; *n* = 6–10/group, ^###^*p* < 0.001 compared with the sham-WT group; *p < 0.05, ***p < 0.001 compared with the Bleomycin-WT group.

To further unravel the role of DOT1L in bleomycin-induced mouse pulmonary fibrosis in vivo, we newly constructed the heterozygous DOT1L-deficient mice (*Dot1l*^+/−^). *Dot1l*^+/−^ mice and its littermate controls (wild-type (WT) mice) showed normal growth under physiological conditions and then we performed bleomycin administration. Interestingly, severe pulmonary fibrosis was shown in bleomycin-administrated WT mice for 14 days but was attenuated in *Dot1l*^+/−^ mice, as determined by H&E staining as well as the Szapiel score (Fig. 4A). In addition, decreased total collagen contents of the lung was observed in *Dot1l*^+/−^ mice, as shown by the progressive decrease in Masson’s trichrome positive staining area compared to the WT group. Besides the pulmonary fibrosis stained by Masson’s trichrome was also further evaluated according to the Ashcroft score (Fig. 4B). Of note, DOT1L depletion in vivo markedly inhibited the protein expression levels of bleomycin-induced pulmonary fibrosis markers (FN, CTGF, α-SMA, Collagen I/III, and MMP2/9; Fig. 4C). Meanwhile, immunohistochemistry staining results also showed that the expression of MMP9 effectively decreased in the lung of bleomycin-induced *Dot1l*^+/−^ mice (Fig. 4D). Furthermore, DOT1L and CTGF both showed significantly increased expression levels in the lung of bleomycin-induced WT mice but decreased expression levels in bleomycin-induced *Dot1l*^+/−^ mice, as detected by the immunofluorescence double staining (Fig. 4E). These above results provide evidences of the in vivo knockdown of DOT1L on reversing bleomycin-induced mouse pulmonary fibrosis.

### Blockade of DOT1L inhibits TGF-β1-induced fibrosis in vitro

**Fig. 5:**
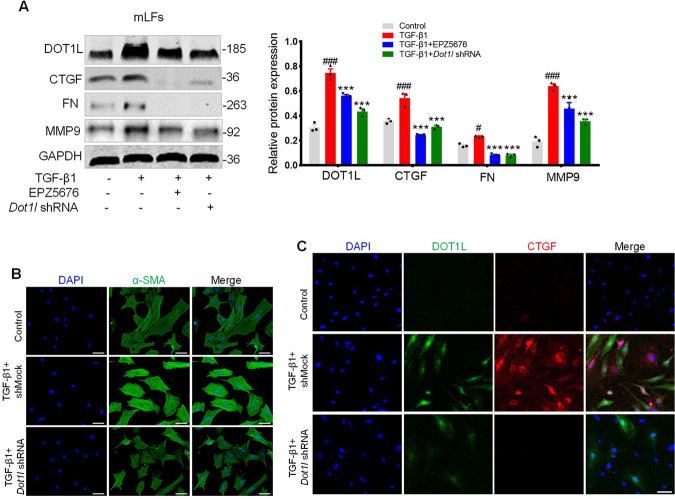
Blockade of DOT1L inhibits TGF-β1-induced fibrosis in vitro. mLFs were transfected with *Dot1l* shRNA for 24 h or pretreated with EPZ5676 (40 μM) for 4 h and subsequently treated with TGF-β1 (10 ng/ml). **A** Immunoblot analysis of DOT1L and lung fibrosis markers (CTGF, FN, and MMP9) in TGF-β1-induced fibrosis after transfecting with *Dot1l* shRNA or pretreated with EPZ5676. All data are presented as mean ± S.D. of three independent experiments. ^#^*p* < 0.05, ^###^*p* < 0.001 compared with the control group; ***p < 0.001 compared with the TGF-β1 group. **B** Immunofluorescence staining of α-SMA, scale bars: 50 μm. **C** Immunofluorescence double staining of DOT1L and CTGF, scale bars: 50 μm.

Next, we sought to determine the impact of DOT1L on TGF-β1-induced fibrosis in vitro. First, we determined the effects of EPZ5676 and *Dot1l* shRNA on TGF-β1-induced mLF fibrosis. As seen in Fig. 5A, knockdown or pharmacological inhibition of DOT1L could block TGF-β1-induced upregulation in the protein levels of lung fibrosis markers, such as CTGF, FN, and MMP9. Moreover, silencing DOT1L decreased the expression of fibrosis marker α-SMA in TGF-β1-induced mLFs, as detected by immunofluorescence staining (Fig. 5B). Meanwhile, the co-expression of DOT1L and CTGF increased upon TGF-β1-induced mLFs but was reversed by DOT1L knockdown (Fig. 5C).

In parallel, we also detected the expression levels of DOT1L and fibrosis markers upon TGF-β1-induced fibrosis in another two different cell lines, including the C3H/10T1/2 Clone8 cells (the mouse fibroblasts) and the WI38 cells (the human fibroblasts). As expected, DOT1L and fibrosis markers including FN, α-SMA, and MMP9 increased in TGF-β1-induced C3H/10T1/2 Clone8 cells (Fig. S1A), while DOT1L-specific inhibitor EPZ5676 downregulated FN, α-SMA, and H3K79me3 levels in TGF-β1-induced C3H/10T1/2 Clone8 cells (Fig. S1B). Similarly, in WI38 cells, we also found that DOT1L and fibrosis markers are increased in response to TGF-β1 while EPZ5676 treatment abrogated the expression levels of Collagen III, MMP9, α-SMA, CTGF, and H3K79me3 (Fig. S1C, D). These above data implicate that blockade of DOT1L inhibits TGF-β1-induced fibrosis in vitro.

### TGF-β1 induces DOT1L upregulation via Smad3 pathway

Smad3 has been well recognized as the major downstream regulator and mediates TGF-β1-induced tissue fibrosis [30]. Hence, we examined whether Smad3 could also be a potential upstream transcription factor of DOT1L in TGF-β1-induced mLF fibrosis. Here we used the Smad3 inhibitor SIS3 to detect the regulatory role of Smad3 on DOT1L. The results showed that inhibition of Smad3 resulted in a decrease on TGF-β1-induced DOT1L protein expression, accompanied by reduced expression levels of fibrosis-related markers, including CTGF, Collagen III, and MMP9 (Fig. S2). These results support that TGF-β1-induced DOT1L upregulation might be mediated by Smad3 pathway.

### DOT1L mediates lung fibrosis at least partly in a Notch-dependent manner

**Fig. 6:**
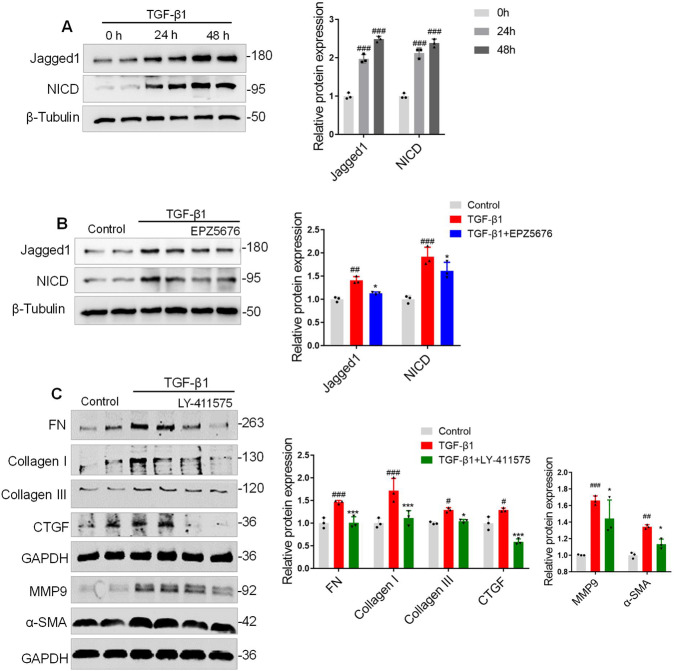
DOT1L mediates fibrosis response via Notch1 pathway in TGF-β1-induced mLFs. **A** Immunoblot analysis of Jagged1 and NICD upon the time-dependent TGF-β1 stimulation. All data are presented as mean ± S.D. of three independent experiments. ^###^*p* < 0.001 compared with 0 h. **B** mLFs were pretreated with DOT1L inhibitor EPZ5676 (40 μM), subsequently incubated with TGF-β1, and Notch signaling (Jagged1-NICD) expression levels were detected by western blot. **C** mLFs were pretreated with Notch signaling inhibitor LY411575 (50 μM), subsequently incubated with TGF-β1, and immunoblot analysis of the fibrosis-related phenotypes (FN, Collagen I/III, CTGF, MMP9, and α-SMA) were detected by western blot. All data are presented as mean ± S.D. of three independent experiments. ^#^*p* < 0.05, ^##^*p* < 0.01, ^###^*p* < 0.001 compared with control; *p < 0.05, ***p < 0.001 compared with TGF-β1.

Notch signaling has been recently shown to be involved in several kinds of tissue fibrosis, including pulmonary fibrosis [31], and also modulates the lung fibroblast phenotypes [26, 32]. However, the relationship between DOT1L and Notch signaling in lung fibrosis has not been explored. Therefore, the expression pattern of the Notch ligand (Jagged1) and the NICD were evaluated in TGF-β1-induced mLFs. We found that Jagged1 and NICD protein levels were upregulated in a time-dependent manner upon TGF-β1 treatment (Fig. 6A), while EPZ5676 inhibited the Notch signaling activation (Fig. 6B). Recent studies have shown that the γ-secretase inhibitor LY411575 significantly attenuated TGF-β1-induced epithelial-to-mesenchymal transition by inhibiting the Notch signaling activation in vitro [33]. To further determine whether DOT1L regulated lung fibrosis in a Notch-dependent manner, mLFs were preincubated with LY411575 for 4 h and then treated with TGF-β1. As shown in Fig. 6C, LY411575 administration significantly decreased the fibrosis-related phenotypes, including FN, Collagen I/III, CTGF, MMP9, and α-SMA.

**Fig. 7:**
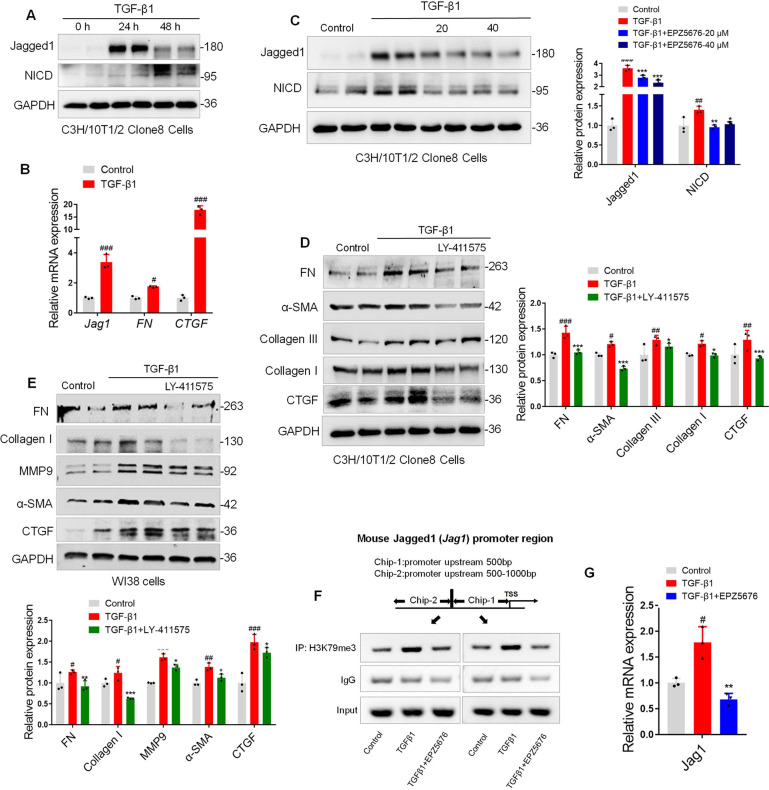
The enrichments of H3K79me3 at *Jag1*’s promoter activate the Notch signaling and accelerate the lung fibrosis. **A** Immunoblot analysis of Jagged1 and NICD upon the time-dependent TGF-β1 stimulation. **B** mRNA levels of *Jag1* and fibrosis marker genes *FN* and *CTGF* increased at 6 h after TGF-β1 treatment; data are presented as mean ± S.D. of three independent experiments. ^#^*p* < 0.05, ^###^*p* < 0.001 compared with the control group; **C** EPZ5676 decreased the Notch signaling (Jagged1-NICD) expression. C3H/10T1/2 Clone8 cells were pretreated with EPZ5676 (20, 40 μM), subsequently treated with TGF-β1, and the expression of Jagged1 and NICD were detected by western blot. **D**, **E** LY411575 downregulated the TGF-β1-induced fibrosis markers both in C3H/10T1/2 Clone8 and in WI38 cells. C3H/10T1/2 Clone8 cells or WI38 cells were pretreated with LY411575 (50 μM), subsequently treated with TGF-β1, and fibrosis markers (FN, Collagen I/III, CTGF, MMP9, and α-SMA) were detected by western blot. **F** ChIP-PCR for enrichments of H3K79me3 at *Jag1*’s promoter in C3H/10T1/2 Clone8 cells. C3H/10T1/2 Clone8 cells were pretreated with EPZ5676 and then incubated with TGF-β1 for 6 h. **G** EPZ5676 inhibited *Jag1* mRNA level in TGF-β1-induced fibrosis. All data are presented as mean ± S.D. of three independent experiments. ^#^*p* < 0.05, ^##^*p* < 0.01, ^###^*p* < 0.001 compared with control; *p < 0.05, **p < 0.01, ***p < 0.001 compared with TGF-β1.

In parallel, we also detected the effect of DOT1L on Notch signaling activation in both C3H/10T1/2 Clone8 cells and the WI38 cells. As expected, Jagged1 and NICD expression levels were profoundly upregulated in TGF-β1-induced C3H/10T1/2 Clone8 cells (Fig. 7A). Accordingly, *Jag1* mRNA level were markedly increased accompanied with the upregulated fibrosis markers FN and CTGF at 6 h in TGF-β1-induced C3H/10T1/2 Clone8 cells (Fig. 7B). Furthermore, EPZ5676 decreased the Notch signaling, as shown by the decreased Jagged1 and NICD expression levels (Fig. 7C). In addition, we found that the Notch signaling inhibitor LY411575 also downregulated the TGF-β1-induced fibrosis markers (FN, Collagen I/III, CTGF, MMP9, and α-SMA) both in C3H/10T1/2 Clone8 cells and in WI38 cells (Fig. 7D, E).

It is well known that DOT1L is the only identified histone methyltransferase that methylate H3K79 and is involved in epigenetic regulation of gene transcriptions. Moreover, we also have demonstrated that the enrichments of H3K79me3 was increased after TGF-β1 administration but reversed by EPZ5676 both in C3H/10T1/2 Clone8 cells and in WI38 cells (Fig. S1). On this basis, to further elucidate whether upregulated *Jag1* mRNA level was due to H3K79me3 enrichments on the Notch ligand Jagged1 (*Jag1*)*’s* promoter region, here we performed the chromatin immunoprecipitation PCR (ChIP-PCR) experiment with anti-H3K79me3. It is interesting to note that H3K79me3 occupancy on the promoter of *Jag1* was markedly upregulated in TGF-β1-induced fibrosis, which was alleviated upon DOT1L inhibition with EPZ5676 (Fig. 7F). Besides, the increased expression of *Jag1* mRNA upon TGF-β1 treatment was inhibited by EPZ5676 (Fig. 7G). These findings described above suggest that TGF-β1-induced DOT1L upregulation might lead to increase the abundance of H3K79 trimethylation at the promoter region of *Jag1*, promoting *Jag1* gene transcription and binding to the Notch1 receptor, further releasing the NICD to translocate into the nucleus, modulating the profibrotic genes expression and accelerating the lung fibrosis.

## Discussion

Pulmonary fibrosis is the advanced stage and fatal cause of many lung diseases, especially IPF. Modulation of lung regeneration and fibrosis could have substantial value in treating lung diseases [34]. However, the specific epigenetic mechanism of pulmonary fibrosis has not been thoroughly studied. In this study, we discovered the direct participation of DOT1L in regulating the pulmonary fibrosis phenotypes, contributing to the development of IPF via Jagged1-Notch signaling. *Dot1l*^+/−^ mice as well as intervention with the DOT1L small-molecular inhibitor EP5676 blunted the pulmonary fibrosis phenotypes including the collagen deposition and increased pulmonary fibrosis marker expression levels. On this basis, the present study discloses a novel role of DOT1L in regulating the pathophysiology of pulmonary fibrosis and inhibition of DOT1L may represent an effective approach to improve the lung functions in the pulmonary fibrosis.

Pulmonary fibrosis can be caused by a variety of factors. Bleomycin, known as the DNA-damaging antibiotic, is the most widely used to induce the pulmonary fibrosis model. Intratracheal administration of bleomycin could directly damage lung epithelial cells and thus mimic the pathological changes in pulmonary fibrosis [35]. This was supported by our results that ECM deposition, alveolar structure disorder, septal thickening, and fibrosis lesions were emerging in lung sections after bleomycin administration. It is also well documented that the histone methyltransferase DOT1L plays a significant role in various pathogenesis processes, such as DNA damage/repair and carcinogenesis [12]. Interestingly, in our present study, we found DOT1L is upregulated in pulmonary fibrosis models both in vivo and in vitro, revealing a potential indication for its role in pulmonary fibrosis progression. Further exploring with the specific inhibitor EPZ5676 or *Dot1l* shRNA demonstrated that DOT1L disruption inhibited bleomycin-induced mouse pulmonary fibrosis in vivo, as indicated by decreased collagen deposition and pulmonary fibrosis marker expression levels. Furthermore, mice treated with EPZ5676 or *Dot1l* shRNA presented lower Ashcroft histopathological grading score and hydroxyproline contents, which were used as the semi-quantitative methods to evaluate the severity of pulmonary fibrosis. Importantly, studies on constitutive DOT1L knockout mice uncover that DOT1L is essential for embryonic development and prenatal hematopoiesis [36]. Given the premature death of homozygous DOT1L knockout mice, *Dot1l*^+/−^ were newly constructed in the present study. *Dot1l*^+/−^ mice showed normal growth under physiological conditions but showed less sensitive to the progression of pulmonary fibrosis. The findings described above provide evidence that DOT1L may serve as a novel target during the pulmonary fibrogenesis.

TGF-β1 is a prototypical pro-fibrotic cytokine that induces myofibroblast activation [37], resulting in poor lung tissue compliance and further aggravating the occurrence of fibrosis. This was supported by our results wherein TGF-β1-induced mLFs (the primary mLFs), C3H/10T1/2 Clone8 cells (the mouse fibroblasts) and the WI38 cells (the human fibroblasts) showed increased protein levels of lung fibrosis markers, while both knockdown or pharmacological inhibition of DOT1L reduced collagen deposition and expression levels of fibrosis markers in response to TGF-β1, supporting that DOT1L regulated the TGF-β1-induced fibrosis in vitro.

Interestingly, subsequent studies identified that both the bleomycin-induced mice pulmonary fibrosis and lung specimens from patients with IPF upregulated the known Notch-related genes [26, 38] and mesenchymal-specific conditional Notch1-deficient mice reduced bleomycin-induced pulmonary fibrosis [39]. Furthermore, recent studies have shown that repeated lung injury also stimulates persistent upregulation of the Notch ligand Jagged1 (encoded by *Jag1*), which in turn stimulates exuberant Notch signaling in perivascular fibroblasts and enhances fibrosis [27]. Thus, Jagged1/Notch signaling was activated and essential for myofibroblast differentiation in the bleomycin model [26]. In keeping with these findings, our study showed that *Jag1* mRNA level were markedly increased in TGF-β1-induced fibrosis. Besides, DOT1L is the sole histone methyltransferase responsible for tri-methylation of histone H3 at lysine 79, which are identified as a marker of active genes [40, 41]. In the present study, we found that the enrichments of H3K79me3 was increased in TGF-β1-induced fibrosis models but reversed by EPZ5676. Furthermore, H3K79me3 occupancy on the promoter of *Jag1* was markedly upregulated and consequently promoted its transcription, which could be alleviated upon DOT1L inhibition with EPZ5676. On the basis of these observations, TGF-β1-induced DOT1L upregulation might lead to increase the abundance of H3K79 trimethylation at the promoter region of the *Jag1*, further binding to the Notch1 receptor and releasing the NICD to translocate into the nucleus, modulating the profibrotic gene expression and accelerating the lung fibrosis.

## Conclusion

Collectively, these data demonstrate that histone methyltransferase DOT1L is a novel epigenetic driver in pulmonary fibrosis, while blockade of DOT1L effectively decreases the pulmonary fibrosis phenotypes. Pharmacological targeting of DOT1L might represent a promising therapeutic approach for human pulmonary fibrosis and other fibrotic diseases.

## Materials and methods

### Materials

Reagent sources are as follows: Bleomycin and DOT1L inhibitor EPZ5676 were purchased from Meilunbio (Meilun Biotechnology, Dalian, China); recombinant human and mouse TGF-β1 proteins were purchased from R&D (R&D Systems, Inc. USA); the Notch signaling inhibitor LY-411575 was purchased from MedChemExpress (HY-50752); the Smad3-specific inhibitor SIS3 was purchased from Sigma (S0447). Antibodies were obtained from the following commercial sources: DOT1L, Collagen I, FN, and MMP9 were purchased from ABclonal (Wuhan, China); MMP2 and Collagen III were purchased from Servicebio (Wuhan, China); α-SMA and Jagged1 was purchased from Cell Signaling Technology (Danvers, MA, USA); NICD and CTGF for western blot was purchased from Abcam (Cambridge, MA, USA); β-Tubulin and GAPDH were purchased from Proteintech (Rosemont, IL, USA); CTGF for immunofluorescence staining was purchased from Santa Cruz Biotechnology (Santa Cruz, CA, USA).

### Generation of heterozygous DOT1L-deficient mice

Since the homozygous global DOT1L gene deletion in mice was lethal, we used the heterozygous DOT1L-deficient mice (*Dot1l*^+/−^) in this study. Briefly, the CRISPR/Cas9 system is an efficient gene-editing method used to generate mice with a conventional knockout allele for DOT1L (DOT1L-KO); a *Streptococcus pyogenes* Cas9 (SpCas9) target site in the conserved exon 1 was selected. The mRNA encoding the Cas9 nuclease and the short guide RNA targeting DOT1L were injected into zygotes from a C57BL/6 background. The genotypes of the generated mice were identified and confirmed by PCR assay using total DNA isolated from tails. The primers DOT1L-P1 and DOT1L-P2 were used to identify the deletion products. The primers used for identification are as follows: M-Dot1L-chk-F2: CATTTCAGGTGGGTCTGCGA; M-Dot1L-chk-R2: CCTATGGTGGCAAGGTGGAG. The first generation was backcrossed into C57BL/6 background and then finally bred into *Dot1l*^+/−^ mice and littermate WT mice. The *Dot1l*^+/−^ mice and littermate WT mice were bred separately for experiments in different cages.

### Mouse model for bleomycin-induced lung fibrosis

The lung fibrosis model was conducted by intratracheal administration of bleomycin (2.5 mg/kg) in randomly grouped C57BL/6 or *Dot1l*^+/−^ mice. Briefly, the mice were anesthetized and instilled bleomycin or saline into the mouse trachea, followed by 1 ml air injection for even distribution.

For in vivo studies with the DOT1L inhibitor, EPZ5676 (20 mg/kg) was administered intraperitoneally, whereas mice in the sham and bleomycin groups were treated with saline, while *Dot1l* shRNA was also used for knockdown of DOT1L in vivo. In brief, the mice were injected with lentiviral particles that carried shRNA targeting DOT1L (*Dot1l* shRNA) both via tail vein prior to bleomycin administration and via intratracheal instillation during the bleomycin administration. The sham and bleomycin model groups were administrated with vector lentiviral particles (shMock). At 7, 14, and 21 days, mice were sacrificed and the lung samples were harvested for further analyses.

### Isolation of primary mLFs

The primary mLFs were isolated from C57BL/6 mice using the tissue explant technique. Briefly, mice lungs were removed and minced into appropriate size, digesting with 0.25% trypsin for 8 min at 37 °C. Then the digested lung tissues were collected and transferred to a culture plate. Seven–10 days later, mLFs were crawled out and verified the purity by immunofluorescence staining with both mesenchymal cell marker Vimentin and smooth muscle cell surface marker α-SMA. Vimentin was positive staining and α-SMA was negative staining. mLFs were used between passages 3 and 6.

### Cell lines

The human lung normal fibroblast cells (WI38) were purchased from Shanghai Zhong Qiao Xin Zhou Biotechnology Co., Ltd., and the C3H/10T1/2 Clone8 mouse fibroblasts were purchased from Procell Life Science & Technology Co., Ltd. (Wuhan, China). Both cell lines were cultured in MEM (containing NEAA) (Shanghai Zhong Qiao Xin Zhou Biotechnology Co., Ltd., ZQ-300) supplemented with 10% fetal bovine serum and antibiotics (100 units/ml penicillin and 100 μg/ml streptomycin) at 37 °C in a 5% CO_2_ and humidified atmosphere according to the vendor’s protocol.

### Small interfering RNA (siRNA) transfection

*Dot1l* siRNA (5’-GCAGAGGCUGUGUGACAAATT-3’) were produced by GenePharma (Shanghai, China) and control siRNA (5’-UUCUCCGAACGUGUCACGUTT-3’) was used as a control for off-target changes in cells. To introduce siRNA into fibroblasts, the fibroblasts were plated on 6-well plates at 30–50% confluence before transfection. Individual siRNA (15 nM), Lipofectamine RNAiMAX, and Opti-MEM were mixed and incubated at room temperature for 5 min. siRNA–Lipofectamine RNAiMAX complexes were added to cells for 24 h and the medium was replaced by fresh serum DMEM medium after transfection. The efficiency of gene knockdown was verified by western blot 72 h post-transfection.

### Lentivirus generation and infection

The specific shRNA of *Dot1l* were purchased from PPL (Shandong University, Jinan, China). Lentivirus generation and infection were performed according to the method described previously [42]. In brief, lentivirus was generated in HEK293T after transfection with lipofectamine 2000 (Thermo Fisher Scientific, Shanghai, China) and plasmids. Lentivirus-containing medium was collected from the culture media 48 h after transfection and added fresh media to the transfected cells and collected them 24 h later again. The two collections of media were combined and virus particles were pelleted by ultracentrifugation (25,000 rpm, 4 °C, 2 h; Beckman Ti70 rotor). Virus particles were then resuspended with phosphate-buffered saline, aliquoted, and flash frozen.

### RNA isolation, cDNA synthesis, and quantitative reverse transcription polymerase chain reaction (qRT-PCR)

Total RNA was isolated by TRIzol Reagent (Takara, TaKaRa Biotechnology, Dalian, China). Total RNA (600 ng) of each sample was reverse-transcribed into cDNA and amplified using a PrimeScript^TM^1st Strand cDNA Synthesis Kit (Takara) according to the manufacturer’s directions. qRT-PCR assay was conducted using the Taq polymerase (Takara). The primer sequences are listed in Table S1.

### Western blotting

The cells were lysed with LDS Sample Buffer (Thermo, Invitrogen, CA, USA) containing 1% protease and phosphatase inhibitor cocktail (Sigma, St Louis, USA) and 5% β-mercapto ethanol. Samples prepared from the lung tissue were lysed with RIPA buffer (Pierce, Rockford, IL, USA). Whole lysate samples were separated by sodium dodecyl sulphate–polyacrylamide gel electrophoresis and blotted to nitrocellulose membrane. After blocking in 5% non-fat milk, the membranes were subjected to incubation using primary antibodies. Protein bands were detected with fluorophore-conjugated secondary antibodies, and detection and analysis were performed with the Odyssey imaging system (LI-COR) and quantified for relative expression levels by Alpha Imager (Alpha Innotech Corp., San Leandro, CA).

### Hydroxyproline assay

Collagen contents in the mouse lungs were measured with a conventional hydroxyproline method using the Hydroxyproline Assay Kit (Nanjing Jiancheng Bioengineering Institute), and the detection was performed according to the manufacturer’s instructions. Absorbance was measured (*λ* = 550 nm). The amount of hydroxyproline in samples was calculated using standard, prepared according to the manufacturer’s protocol (Nanjing Jiancheng Bioengineering Institute).

### Histological and morphometric analysis

After post-injury, the lung sections of mice were harvested and fixed with 4% paraformaldehyde. The lung tissues were paraffin-embedded and then cut into 4 μm sections to be used as lung slides. For morphometric analysis, the sections were stained with Masson’s trichrome and H&E after deparaffinization and rehydration. Picture acquisition was performed using an automatic slide scanner microscope. The level of lung fibrosis was determined using the Image Pro Plus software (version 6.0, Media Cybernetics).

### Immunohistochemical staining

The lung slides were prepared as described above. Endogenous peroxidases activity was quenched by incubation with dilute hydrogen peroxide (1:10 dilution of 3% hydrogen peroxide). The lung sections were incubated overnight at 4 °C with the corresponding primary antibodies, then the secondary antibody biotinylated anti-rabbit IgG was applied for 30 min at room temperature. The sections were visualized by 3, 3′-diaminobenzidine tetrahydrochloride.

### Immunofluorescence staining

The lung sections and cell slides were fixed with 4% paraformaldehyde and then permeabilized using 0.25% Triton X-100. Then 10% goat serum was used to block the slides for 0.5 h and then incubated overnight with primary antibodies at 4 °C. Appropriate secondary antibodies were added and incubated with the slides for 1.5 h at room temperature. The nuclei were stained with 4′,6-diamidino-2-phenylindole. The images were captured by using a fluorescence microscope (Zeiss LSM780, Carl Zeiss).

### Chromatin immunoprecipitation PCR

ChIP assays of C3H/10T1/2 Clone8 cells induced with TGF-β1 were performed to investigate the interactions between proteins and genes. Briefly, the cells crosslinked with 1% formaldehyde for 15 min at room temperature and then collected in lysis buffer, followed by sonication to shear to 300–1000 bp and then immunoprecipitated with anti-H3K79me3. The same amount of non-specific lgG was used as negative control. Immunoprecipitated protein–DNA complex was purified, followed by capture with protein-G beads. Then the ChIP-DNA was purified and subjected to PCR analysis on *Jag1* gene promoter. Primer sequences are listed in Table S2.

### Statistical analysis

The GraphPad Prism 7.0 software was used for statistical analysis. Experimental results are expressed as mean ± S.D. Differences of means were analyzed by using one-way analysis of variance with Turkey–Kramer post hoc test for multiple groups and unpaired Student’s *t* test for two groups. Probability values *p* < 0.05 were considered statistically significant.

## Supplementary information

Supplemental Material

## Data Availability

All data generated or analyzed during this study are included in this published article [and its Supplementary Information files].
