# Correction: The histone methyltransferase DOT1L is a new epigenetic regulator of pulmonary fibrosis

**DOI:** 10.1038/s41419-022-04548-8

**Published:** 2022-02-14

**Authors:** Di Yang, Peng Xu, Haibi Su, Wen Zhong, Jie Xu, Zhenghua Su, Xinhua Liu

**Affiliations:** 1 Pharmacophenomics Laboratory, Human Phenome Institute, Fudan University, 201203 Shanghai, China; 2 Department of Pharmacy, Taihe Hospital, Hubei University of Medicine, Shiyan, Hubei China

**Keywords:** Epigenetics, Respiratory tract diseases

Correction to: *Cell Death and Disease* 10.1038/s41419-021-04365-5, published online 17 January 2022

The original version of this article unfortunately contained a mistake in figures 1 and 2. The authors apologize for the mistake. The original article has been corrected.

