# Supplementary material for: The histone methyltransferase DOT1L is a new epigenetic regulator of pulmonary fibrosis
Source: Cell Death Dis. 2022 Jan 17;13(1):60. doi: 10.1038/s41419-021-04365-5 (PMC8763868; doi:10.1038/s41419-021-04365-5)
Supplement: Supplementary file 1 — Supplemental Material [file 41419_2021_4365_MOESM1_ESM.docx]

**SUPPLEMENTAL MATERIALS**

**Supplemental Figures**

**Figure S1.**


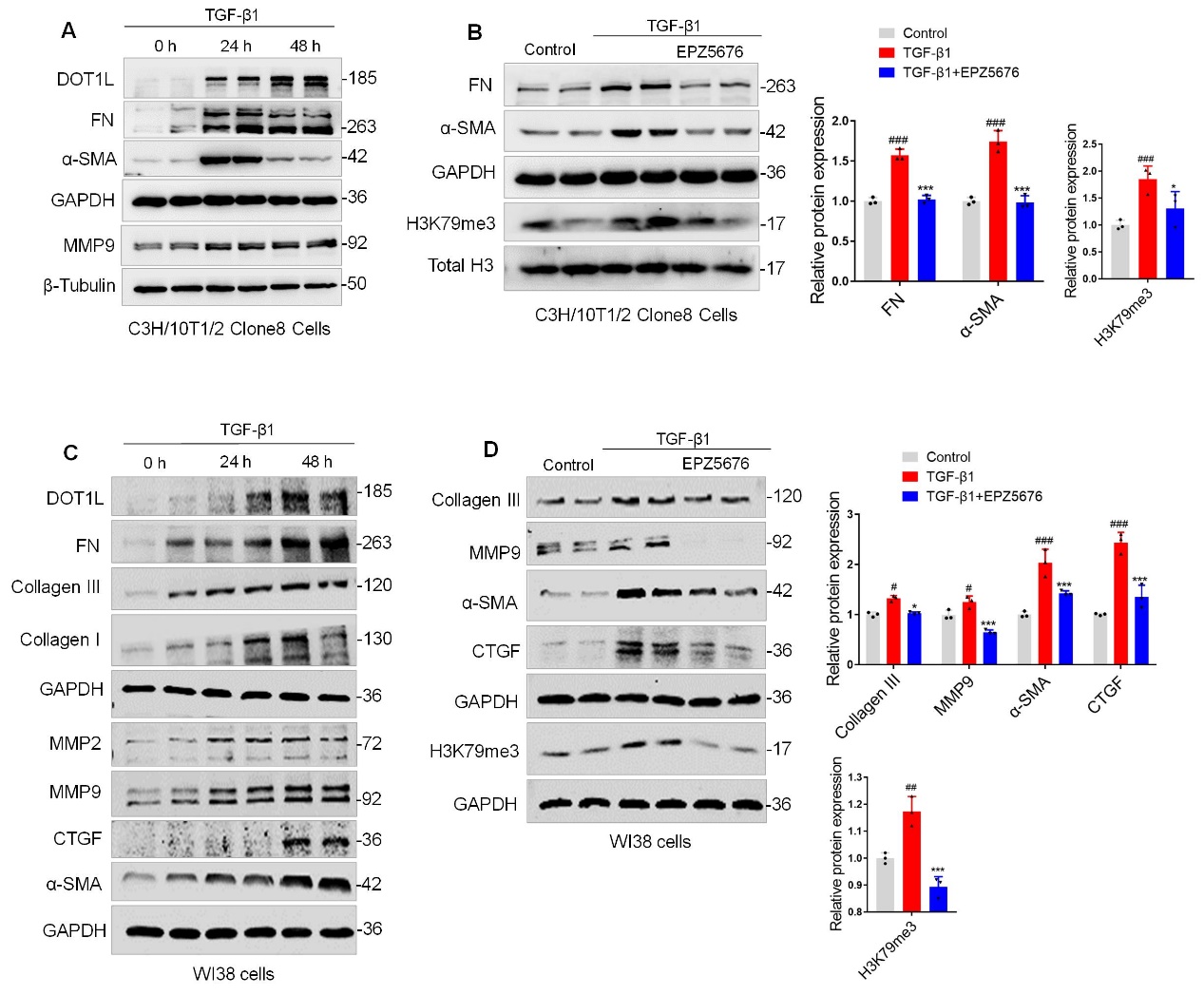


**Figure S1.** **DOT1L increases in TGF-β1 induced fibrosis in both C3H/10T1/2 Clone8 cells and WI38 cells.** (**A**) Western blot analysis for DOT1L and fibrosis markers (FN, α-SMA and MMP9) in TGF-β1 induced C3H/10T1/2 Clone8 cells. (**B**) EPZ5676 treatment reversed the levels of H3K79me3 and fibrosis markers (FN and α-SMA) in TGF-β- induced C3H/10T1/2 Clone8 cells. (**C**) Immunoblot analysis for DOT1L and fibrosis markers (FN, Collagen I/III, MMP2/9, CTGF and α-SMA) in TGF-β1-induced WI38 cells. (**D**) EPZ5676 treatment reversed the levels of H3K79me3 and fibrosis markers (Collagen III, MMP9 and α-SMA) expressions in TGF-β1-induced WI38 cells. Data are presented as mean ± S.D of three independent experiments, ^#^*p* < 0.05, ^##^*p* < 0.01, ^###^*p* < 0.001 compared with control group; ^*^*p* < 0.05, ^***^*p* < 0.001 compared with TGF-β1 group.


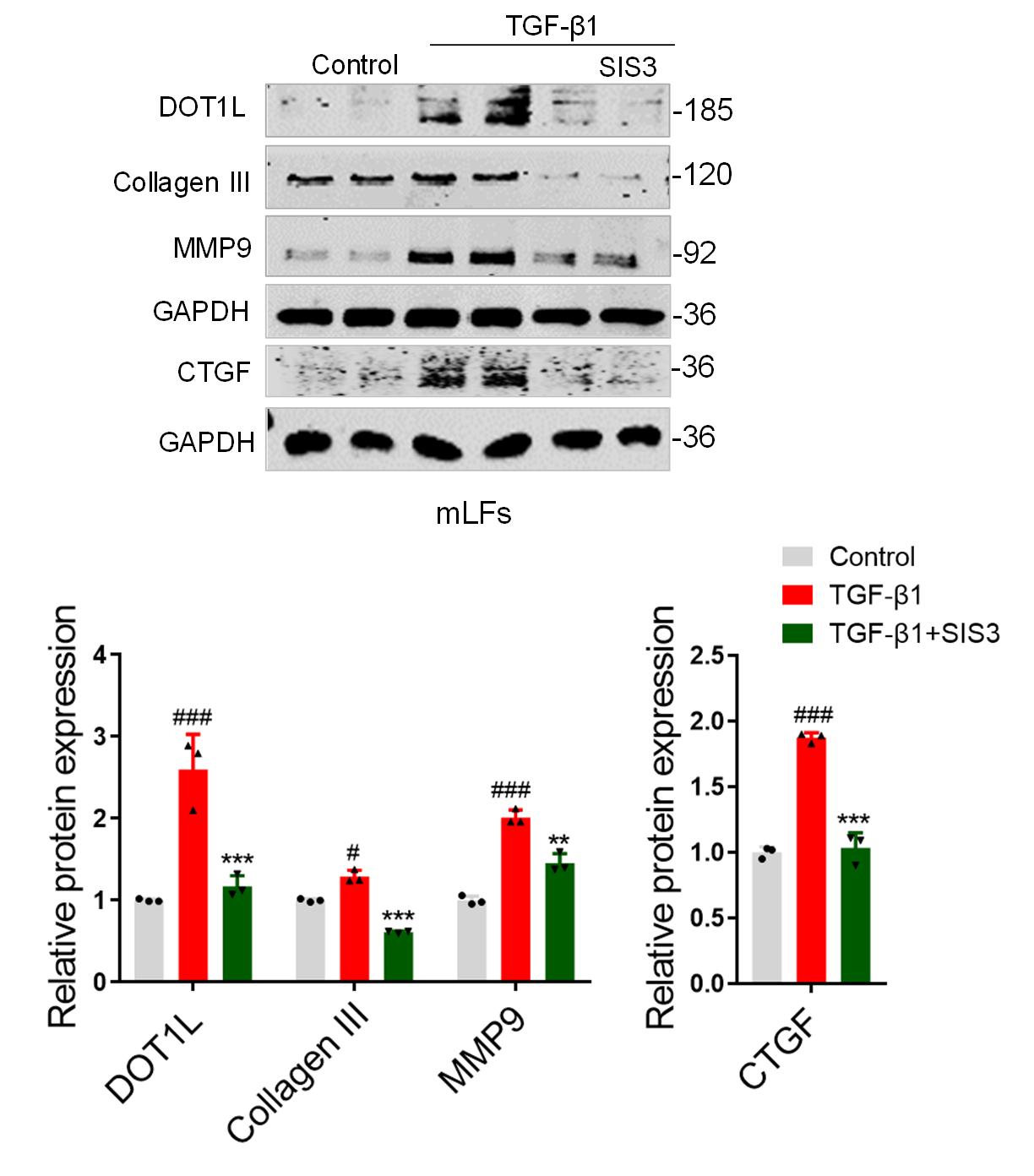


**Figure S2.** **DOT1L increases in TGF-β1 induced fibrosis via smad3 pathway in mLFs.** mLFs were pre-treated with Smad3 inhibitor SIS3 (5 μM) and then induced by TGF-β1. DOT1L and fibrosis markers (Collagen III, MMP9, and CTGF) were determined by western blot. Data are presented as mean ± S.D, n=3, ^#^*p* < 0.05, ^###^*p* < 0.001 compared with control group; ^**^*p* < 0.01, ^***^*p* < 0.001 compared with TGF-β1 group.

**Supplemental Tables**

**Table S1. Primers used for qRT-PCR validation.**

| Primer name | Primer sequence (5´ to 3´) |
| --- | --- |
| Mouse_*DOT1L*_F | CCATCACCAGCCTACCTGTTA |
| Mouse_*DOT1L*_R | AAGCACCAGTCTGCTTCTCAA |
| Mouse_*GAPDH*_F | CCAATGTGTCCGTCGTGGATC |
| Mouse_GAPDH_R | GCTTCACCACCTTCTTGATGTC |
| Human_*DOT1L*_F | GAGACCTCCTTCGACCTGGT |
| Human_*DOT1L*_R | CGACGCCATAGTGATGTTTGC |
| Human_*GAPDH*_F | CTGGGCTACACTGAGCACC |
| Human_*GAPDH*_R | AAGTGGTCGTTGAGGGCAATG |
| Mouse_ *Jag1*_F | TACTGGTGTGTAAGGAAGCGG |
| Mouse_ *Jag1*_R | TCCTCCTCCACTTCCGAGTT |
| Mouse_ *FN*_F | AATCGTGCAGCCTCAATCCC |
| Mouse_ *FN*_R | CAGGCTTGCTCTCGCAGTTA |
| Mouse_ *CTGF*_F | ACCCAACTATGATGCGAGCC |
| Mouse_ *CTGF*_R | TGCACACCCCGCAGAACTT |

**Table S2. Primers used for ChIP-PCR validation**

| Primer name | Primer sequence (5´ to 3´) |
| --- | --- |
| mouse-*Jag1*_TSS_upstream 500bp_F | CGGTCCTTCCAGGTTCCTTTC |
| mouse-*Jag1*_TSS_upstream 500bp_R | GGCCACCTCTTCTCAACACG |
| mouse-*Jag1*_TSS_upstream 500-1000bp_F | GGCAAGTCTGACTCCGGGAA |
| mouse-*Jag1*_TSS_upstream 500-1000bp_R | CCTTGCAAGCCCCAGGTGTA |
